# Loot

**Published:** 1870-10

**Authors:** 


### Loot.

***Chloral-iana.*** [From *American Eclectic et al.*]

Dr. T. S. Clousten, Medical Superintendent of the Cumberland and Westmoreland Asylum, thus sums up his experience after having given the hydrate of chloral in forty cases of various forms of insanity:

"1. It has proved a most safe and certain sleep-producer. It seems certain that by it we can compel sleep in any case.

"2. By means of this property, attacks of insanity may probably be warded off in some cases.

"3. Its action in abating and soothing excitement is more uncertain than its sleep-producing power, and lasts a shorter time than that of any signally powerful drug; but it is most valuable in certain cases, especially in some recent and curable ones, where formerly we should have been afraid to give opium. It has no directly curative action, but it evidently could be so employed as to tide over short attacks of insanity, and to prevent certain cases from being sent to lunatic asylums.

"4. Whether it does good or not, it never does harm. In this respect it is the very king of all narcotics.

"5. Its effect on the temperature of the body is variable in different cases, and in the same case at different times; but generally it is to reduce the temperature slightly, taking the average of a number of patients. It differs from opium in this respect, which raises the temperature; but the reduction caused by chloral is not nearly as great in maniacal excitement as that caused by alcohol in large doses.

"6. It should be given to subdue brain excitement in doses, beginning at twenty or thirty grains, repeated from three to five hours. To produce sleep in great excitement from forty to sixty grains are required, the latter dose not failing in one per cent. of the cases."—*British Med. Journal*, May 7, 1870.

Dr. John B. Tuke, Medical Superintendent of the Fife and Kinros District Asylum, has employed chloral with good results in acute mania, asthenic insanity, the insomnia of melancholy, and in chronic cases of insanity, in which violent outbursts of excitement occur. "The advantages of chloral," he says, "over all our hypnotics with which I am acquainted, are—

"1. That it is more uniformly certain in its action.

"2. That it has no depressing influence.

"3. That it does not cause constipation.

"4. That it does not produce nausea.

"5. That its effects are more lasting.

"I believe it to be the most valuable means of procuring sleep which has yet been introduced into the Pharmacopœia of the asylum physician."—*Lancet*, March 26, 1870.

Mr. Spencer Wells stated, at a meeting of the Obstetrical Society of London, that in a case of furious maniacal excitement seen by him with Dr. Munro, one thirty-grain dose of chloral was followed by almost immediate calm and afterward sleep.—*Lancet*, April 2, 1870.

Dr. Playfair also reported to the same society a case of threatened puerperal mania, where the patient had become maniacal after a previous labor, and after the present one exhibited the same symptoms which had preceded the previous attack, viz: restlessness, inability to sleep, etc. Thirty grains of chloral given at bedtime produced a long and quiet sleep, and the same dose was repeated every night for a week. Dr. P. has no doubt that this medicine kept off the threatened attack.

A case of puerperal mania was communicated to the Obstetrical Society of Edinburgh, by Dr. Thompson, who stated that from his observation in that case he looked forward to chloral being of the greatest service in the acute stage of that disease.—*Edin. Med. Journal*, May, 1870.

Mr. R. G. Hill reports (*Med. Times and Gazette*, April 9, 1870,) a case of acute mania in a female, in which the usual remedies were tried without benefit, when chloral was given with the happiest effect.

Much additional testimony has been adduced as to the value of the hydrate of chloral in *delirium tremens.* According to the experience of the physicians of the Royal Infirmary, Edinburgh, chloral seems in this disease "to be almost a curative agent—as in most cases, notwithstanding violent excitement or delirium, it produces a sound sleep, from which the patient frequently awakes sane and rational. In doses of forty or sixty grains, repeated every half hour three or four times, a deep and lengthened sleep generally ensues. Although there are several exceptions, many most interesting and remarkable cases might be cited to prove the general rule."—*Brit. Med. Journal*, April 30, 1870.

Mr. Maunder has employed it successfully at the London Hospital, in a case of furious delirium tremens in a woman. A dram dose was given, and within half an hour she was fast asleep, all maniacal symptoms being abolished for the time. The dose was repeated, with the effect of keeping her quiet, and at the date of the report she seemed quite rational.—*Brit. Med. Journal*, April 2, 1870.

Dr. George W. Balfour has been equally successful with chloral at the Royal Infirmary, Edinburgh. He relates six cases of delirium tremens successfully treated, and says that the cases in his wards "vary from the merely excited fidgetty condition, known as the horrors, to the most exaggerated mania, often accompanied by repeated epileptiform convulsions. The cases given are amongst the severer ones treated; the milder ones succumbed more readily to the treatment, one dose being usually sufficient; the general result being to keep the wards almost empty, from the rapidity with which the patients are enabled to be discharged."

Dr. C. A. Stivers reports (*Pacific Med. and Surg. Journal*, May, 1870), two cases of delirium tremens successfully treated with chloral, in the San Francisco Hospital.—*Journal of Med. Sciences.*

The late Dr. J. Y. Simpson (*Medical Times and Gazette*), in his "Therapeutic notes on Chloral," remarked as follows: "I am not aware of any special contra-indications to the employment of chloral when used for somniferous purposes. Even in head and chest affections, when I should have been chary of having recourse to opium as an hypnotic, I have employed chloral with perfect success. The contra-indications to opium offered by a tendency to constipation, etc., do not exist against chloral."—*Med. Record.*

M. M. Pallen, M.D., St. Louis, Mo. (*The St. Louis Med. and Surg. Journal*), coincides with M. Demarquay that Dr. Liebreich's assertion "that chloral is decomposed in the blood, and that the chloroform resulting from this decomposition produces anæsthesia," is not the correct view. If chloral acts as an hypnotic and as a hyperæsthetic, it cannot be *totally* converted into chloroform. There is no doubt in his mind that chloroform is produced during the action of the chloral, but the chloroform arises chiefly by oxydation in the lungs. Dr. P. coincides with those who think that it is an hypnotic, and valuable as such an agent where opium cannot be used. He has used it extensively in monomania, endometritis, and puerperal convulsions, to procure sleep; in some instances as high as a drachm; to children, in doses from six to twenty grains, according to age.

It is applicable in that form of affection known as night-terrors. The child is sleepless, or even when it sleeps the slumber is disturbed, and it moans or it grits the teeth. All this should be overcome, or else the child in after years will be an epileptic. Chloral is the remedy, opium the poison. The dose to children will vary from four to twelve or more grains. He always uses, as the vehicle with which to mix the chloral, the syrup of tolu.—*Med. Record.*

***Snake Poison and its Antidote.***

The following communication appears in a recent issue of the *European Mail*, and throws an important new light on the therapeutics of animal poisons:

Sir—Having noticed of late the publication in both European and American journals of articles upon the subject, and particularly one under date March 2, 1870, under the heading, "The Cobra Question in India," I trust you will give publicity to this communication, on account of its importance; and am induced to ask for it a place in the columns of your journal, in the hope that it will afford to your readers, in India more particularly, a knowledge of an antidote for snake poisons, which may claim to be specific, insomuch as it has never been known to fail in a single instance during the past three years in different districts in this country, in which I have been able to induce its general adoption, and particularly by the *curanderos*, or curers (snake charmers). I have devoted no little time during the past twenty years to a study of the habits, peculiarities, etc., of poisonous snakes, and have made many experiments with their poisons, with a view to discover, if possible, specific antidotes to them, and have been so far successful as to be able to announce the law in therapeutics that "all animal poisons have their specific antidotes in the gall of the animal or reptile in which these poisons exist."

The bite of the *cobra*, or of any other poisonous snake or reptile, can be cured by administering a few drops of a preparation of the gall of the *cobra*, which should be prepared as follows: Pure spirits of wine, or 95 per cent. alcohol, or the best high wines that can be procured, 200 drops; of the pure gall, 20 drops; in a clean two-ounce phial, corked with a new cork; give the phial 150 or 200 shakes, so that the gall may be thoroughly mixed with the spirits, and the preparation is ready for use. In case of a bite put five drops (no more) of the preparation into half a tumblerful of pure water; pour the water from one tumbler into another, backwards and forwards several times, that the preparation may be thoroughly mixed with the water, and administer a large tablespoonful of the mixture every three or five minutes until the whole has been given. In case the violence of the pain and hæmorrhage or swelling of the bitten part should be but slightly alleviated after the whole has been taken, repeat the dose, prepared with the same quantity of the preparation in the same way, and administer as before. In curing upwards of fifty cases of snake bites I have never been obliged to repeat the dose except in two instances, and have never lost a case. The *cobra* poison is no more deadly than that of a great variety of snakes found in South America, of which may be named the *Cascabel*, or *Rattlesnake; Boqui-dorada*, or gilded mouth; *Mapana-sapo*, or frog-headed Mapana; *Mapana-fina*, or *Lachesis, Niger*, *Birri* and *Verrugosa*, or wart snake. The poison of all these varieties produces death (under certain conditions—atmospherical, physical, climaterical, and electrical) in from fifteen minutes to two or three hours; but it is found that the gall of each variety (administered as previously indicated) is the perfect antidote for its own poison. The gall of the most deadly kind may be used in cases of bites of those less virulent, and is also applicable in cases of bites of the centipede, scorpion, stingray, star-lizard, or *Lacerta-stella*, and is also very effective in dog-bites. The native curers use a tincture of a plant called *Alconcito*, or *solobasta*, for bites of the *Cascabel* and *Boqui-dorada*, with very good success in cases of bites, and also as a prophylactic, by inoculation (in the point of the shoulder), for preserving themselves harmless against these poisons. For this purpose incisions are made at the lower point of attachment of the deltoid muscles, in the same manner as for vaccination, and into these are introduced small pellets of cotton (of the size of a millet seed) saturated with an alcoholic tincture of the *Alconcito*. Care is taken to keep within doors and out of the wet and dew for from fifteen to twenty days, after which period the inoculation is concluded. Of the efficacy of this process, I can say that I have repeatedly tested it on dogs, in a district where every dog not inoculated, if bitten by a snake, invariably dies, and have never known an inoculated dog to show any inconvenience from the bite of the most venomous viper. This plant is the *Aristoloquia Colombiana.* In Brazil, the curers use the tincture of the *Aristoloquia milhomeus*, or *Arist. grandifloras.* In the United States, the Indians use the *Serpentaria*, or *Aristoloquia Virginiana*, and it is more than probable that the *Arist. Colo.*, or the *grandifloras*, is to be found in India.

During my research in this branch of natural history I have collected much interesting and valuable information, all of which I have incorporated in a small work that will shortly be published in English; but the reports of such a frightful number of deaths from snake bites as English journals record as having occurred during the past year in certain parts of India, have led me to address this letter to you, that the truth of the efficacy of this antidote for snake bites may be tested by every person who takes any interest in the matter, and that these tests may be so effectually made that a point of such vital importance as the discovery of the specific antidote for these poisons may be known throughout the world.

I indulge the hope that I may see repeated corroborations of the results of my own humble labors in this specialty through so many years.

Your obedient servant,

S. B. Higgins.

*State of Magdalena*, April 10, 1870.

—*Chem. & Drug.*, *Lond.*, June 15, 1870.

—*Am. Jour. Pharmacy.*

### *A Dangerous Water-Pipe.*

Attention has been called several times in this journal to the dangerous character of the *galvanized iron pipe*, when employed for conducting water to be used for culinary purposes. Instances of severe poisoning from the use of this pipe are continually coming to our notice, and we are led once more to caution our readers against it. It is almost a crime for dealers and manufacturers to recommend this zinc-covered iron pipe for water conduit, as they thereby jeopardize the health and perhaps the lives of purchasers. When this pipe comes from the hands of the manufacturers, it has a fresh, clean appearance, and to those who do not understand the nature of the covering, the idea is conveyed that it will not oxydize or rust, like ordinary iron pipes. But this is an error; it will rust even more rapidly than clean iron in most localities. The superficial covering of zinc is rapidly decomposed under the influence of ordinary pond and spring waters, and the oxide, carbonate, and chloride of zinc are formed, which salts are of a deleterious or poisonous character. This covering of zinc on the interior is attacked immediately when water is allowed to flow through, and in some instances we have known it to be entirely removed in forty-eight hours. The insoluble carbonate of zinc is seen to float upon the water in the tea kettle and other water vessels used in families, and this has often created alarm where no suspicions previously existed. We hope the newspaper press throughout the country will caution their readers against the use of this pipe for water supply.—*Boston Journal of Chemistry.*

### *Therapeutic Uses of Iodoform.*

Dr. Stiles Kennedy, of Newark, Delaware, communicates the results of his observations on the effects of this drug, which he considers to possess great value in various constitutional and nervous diseases. He gives the details of a case of periodic gastric neuralgia, probably connected with a rheumatic diathesis of several months' standing, in a man not 30 years of age, of antecedent good health and vigor. The pains in the stomach recurred on each afternoon, and lasted well during the remainder of the twenty-four hours. Quinine, iron, morphia, arsenic, blisters, mercury, and other remedies, had been administered without advantage. Two grains of iodoform, and the same quantity of pulv. ferri, were prescribed in pills three times a day, and in a week he was well. The second case was that of a man aged 45, who suffered from violent and frequently recurring pain in the scalp, running from the eyebrows to the occiput. A great variety of treatment had here also been adopted, the last being heroic doses of quinia, morphia, iron, and arsenic in combination, but without effect. Dr. Kennedy therefore ordered iodoform, pulv. ferri, of each one hundred grains, veratria one grain; to be divided into fifty pills, one to be taken three times a day. After twelve days' continuance the pain completely disappeared, and in the course of the last year only recurred twice, on each occasion being quickly removed by recommencing the use of the pills. A similar combination, without the veratria, was found very effective in sciatica, by another physician, and in several cases of ague by a third. Dr. Kennedy recommends it strongly as an addition to the ordinary plasters and ointments for syphilitic periostitis, etc.; in fact, he says an ointment containing from thirty to sixty grains of iodoform to an ounce of lard, is one of the most delightful remedies, so far as relief is concerned, to painful burns, sores, chancres, boils, etc., that can be found, promoting rapid healing. In two cases of chancre the dry powder was applied, with magical results.—*The Medical and Surgical Reporter.*

***Fistula in Ano.*** By Tom O. Edwards, M.D., Lancaster, O.

In July number, 1870, of the *Gynæcological Journal*, (Boston), I find ingenious methods of performing the operation for above; and as any mode in some cases (so obnoxious to many patients is the knife,) that would be a success, and, indeed, in this, as in all cases, the more simple, if successful, is the best, I send you the result of my operations. In one case nothing but the knife could have been successful, as it was of twenty years duration, and the patient was induced to pound the buttocks and perineum with a small club, with a view of producing inflammation and union. This resulted in four fistulas, all radiating from one, and after the operation the admeasurement of the various cuts through the buttocks was nearly seventeen inches. This is possibly the largest on record, and was entirely successful, by careful dressing and slow healing.

What I propose to recommend in simple fistula, is the use of the string, differently introduced from the above cited cases, and a different string. The common silk ligature will rot in cases where there is much delay, and the shortest time employed by me has been twenty days. Having been disappointed, and, in one or two cases nearly well, finding the string to give way on being tightened, and thus giving the trouble of introducing another, in order to complete the cure, I resorted to a string made of sea grass, knowing its ability to bear any quantity of water any length of time without rotting, and very strong. My process is this: Introduce into the fistula* a canula, and immediately thereafter the index finger of the left hand in the rectum, feeling the canula, having previously prepared a silver or iron wire annealed, to the end of which is tied the sea-grass string; introduce the free end of the wire through the canula, push it forward, and with the finger in ano, bend the wire down until it protrudes through the sphincter, then draw out until the string is in proper position. This is better than the probe with an eye, as you could not readily disengage the wire from the eye. After that the process is similar. You need no anal speculum. This sea-grass string is rougher and a better cutter—arouses more inflammation than silk or linen, and the one I use I could use a dozen times without rotting. I write this because I see drawings of speculums, needles, and the parts engaged in the disease, in the journals, and knowing my plan is more simple, and easier adopted, I am induced to give it publicity.

The treatment of fistula in ano by ligature was the foundation of the reputation of a "quack," in Louisville, that should have

* If incomplete fistula, make it complete by an exploring needle or other sharp instrument.

been undermined by the surgeons there in six months. All that is needed is the introduction of the string; after that, the patient can daily gradually tighten it. I can most confidently recommend this mode of operating, and in cases of tuberculosis, as the cure is so slow, is accompanied by the usual discharge, and there is no danger from its repression, and needs only patience and attention on the part of the patient. I seldom anæsthize the patient, so rapidly and painlessly is the operation performed.—*Leavenworth Med. Herald.*

### *A New Remedy for Asthma.*

The following extract from a letter lately received, makes mention of an addition to the *materia medica:* "Dr. P., of Lawrenceburg, Indiana, some forty years ago related to me, among other incidents of early frontier life on the Ohio, his experience of a novel remedy involuntarily taken. The doctor had from childhood been a martyr to asthma; it was the rock ahead for which he was continually on the lookout. One day—it must now be near seventy years ago—in traveling the rounds of a country practice, and while yet at a distance from any shelter, a storm of rain burst suddenly upon him. He rode at full speed for the nearest cabin, distant some miles, and reaching there, drenched to the skin and gasping for breath, he dismounted and made the best of his way to the door. As he entered, he met the inmates of the cabin rushing pell-mell out into the rain, holding their noses, and giving expression in every possible way to the most extreme disgust. He was not long in discovering the cause for their actions. The dogs had chased a polecat under the floor, and the whole atmosphere was loaded with its horrid effluvium. It was a choice between polecat and asthma, and Dr. P. chose the former and remained in the house. To his surprise the constriction of his chest began to disappear with the first inhalation, and in ten minutes he was free from every trace of the paroxysm. As soon as he could do so, he procured the musk-bag of one of the animals, and prepared an alcoholic tincture. The scent of this never failed to avert promptly a paroxysm of the disease; and ultimately the remedy, or time, or both combined, effected a permanent cure. He assured me also that he had successfully used it in similar cases to his own. Belonging, as it does, to the same class with musk and castor, but infinitely more active and efficient, if its odor be taken as a criterion of power, it may prove a valuable antispasmodic. I have seen cases of hysteria upon which, from a safe distance, I should have been delighted to witness a trial of its powers."—*American Practitioner.*

***Cinchona Cultivation in India.***

C. B. Clarke, Esq., officiating superintendent of the botanic gardens, and in charge of cinchona cultivation in Bengal, in his annual report to the secretary of the government of Bengal, makes some interesting statements relative to cinchona cultivation in India. Mr. Clarke thinks that cinchonas thrive best about two thousand feet above the ocean, and grow very well down to the rivers at eight hundred feet elevation. *Cinchona calisaya* grows best at similar elevations, but will not grow so high as *C. succirubra.* The only plants in the Rungbee plantations which are *above* four thousand feet elevation, are some *C. officinalis*, which are being tried at this higher level as a last experiment as to whether the species can be cultivated at all at Rungbee.

The manufacture of the crystallized sulphate of quinine now in general use, consists of two stages. The bark is treated with dilute acid, which takes up the alkaloids contained in it, and also a certain quantity of coloring matter, resin, and other rubbish, which quantity is considerable in *C. succirubra* bark, the only bark which for some years will be produeed in quantity at Rungbee. The acid infusion is neutralized by an alkali, when the quinine alkaloids are precipitated, accompanied with more or less of the rubbish. These precipitated alkaloids (more or less impure) are known as the first stage of the manufacture. The second stage is, in actual manufacture, generally performed by washing the quinine out of these precipitated alkaloids by strong alcohol, and evaporating the alcohol.

According to Dr. Broughton's cinchona report of August, 1868, the quinine alkaloids are valuable febrifuges, and operate pretty much as quinine, but are less powerful. Roughly, it may be said that two grains of cinchonidine are equal in all respects to one grain of quinine. The action of cinchonidine and the other minor quinine alkaloids is less satisfactorily made out, but in the *C. succirubra* bark usually 75 per cent. of the alkaloids present are either quinine or cinchonidine. If the precipitated alkaloids are dissolved in dilute sulphuric acid, the dose is equivalent to that of the crystallized sulphate diluted in acid. Several doctors of experience in the medical department of the government object that the crystallized sulphate is pure, and that they can be sure of the dose they give to a grain; whereas in the precipitated alkaloids the proportions of cinchonidine, and even more inert alkaloids, will be variable and doubtful.

Cinchonidine is about one-third the value of quinine, and its crystals are exceedingly like those of quinine. There is very little quinine in general use at present that is not deeply adulterated with cinchonidine. Should the production of precipitated alkaloids be carried on upon a large scale, it would be possible to thoroughly mix a large quantity, analyze a sample, and then issue it with the percentage of quinine and cinchonidine certified on the labels of the bottles.

After protracted experiments, Mr. Clarke states it as his opinion the successful manufacture of quinine is not a particular secret, or the adoption of any particular routine, but is the result of the application of skillful manipulation and minute experience at each step and turn of the work.

The prunings and thinnings of this season are estimated to produce about 1,500 pounds of bark, of which nearly 1,000 pounds will be bark from wood three or four years old, the remainder from second year bark.

In the bark, from very young shoots, the quantity of resin present is large, and it must be dealt with by boiling in alkaloid water, notwithstanding the sacrifice of a percentage of the small quantity of quinine present, consequent upon the operation.

In regard to working fresh bark, Mr. Clarke says:

It has been stated that bark gives up its quinine more readily in a fresh state. I have tried the fresh bark and have not obtained a good result. I have little doubt that this failure was because my bark was not cut fine enough. Indeed, I could see that the dilute acid discolors the bark hardly one-fortieth of an inch deep. There will always be a great practical difficulty in working the fresh bark on a large scale. The fresh bark cannot safely be stored, as it soon ferments. Now the bark only "rises," that is, is fit for stripping, at certain times; so that in the factory, if we worked fresh, we should be compelled to provide a very large quantity of machinery indeed, so as to be able to take in hand, at once, the bark that would come in upon us irregularly in masses. By working it dry we can work it regularly at our leisure.

The number and distribution of cinchona plants in the government plantations at Darjeeling on the 1st of April, 1870, are stated as follows: Of all species, number in permanent plantations, 1,500,658; number of stock plants for propagation, 40,000; number of seedlings or rooted cuttings, in nursery beds for permanent plantations, 379,325; number of rooted plants in cutting beds, 340,127; number of cuttings made during the month, 2,000; total number of plants, cuttings, and seedlings, 2,262,110.—*Dep. Agriculture, July*, 1870.

### *Efficacy of Large Draughts of Pure Water in Poisoning.*

The following note, taken from the works of Sydenham, is full of instruction: —

"I was called to a servant who had taken a large dose of corrosive sublimate. About an hour had elapsed when I arrived. His mouth and lips were already much swollen; he complained of violent pain and burning heat in the stomach, and was extremely ill. I ordered him at once to drink at intervals, but as speedily as possible, twelve pints of tepid water, and that as often as he should vomit he should begin to drink more. I ordered also that, as soon as it should be perceived by the pains of the bowels that the poison was gaining the bowels, he should be given a number of injections of simply tepid water. The patient did as was ordered, and drank even a greater number of pints of water than ordered. The first vomits were extremely acrid, on account of the quantity of corrosive sublimate which they contained; those which he threw up successively, became less acrid with each successive ejection, until they became completely free. The colics which came on were tempered by tepid water clysters. This simple means was so successful that in a few hours the patient was out of danger. There remained only swellings of the lips and excoriations of the mouth, caused by the acridity of the poison contained in the water which he threw up; but by the use of milk, which I had him take as a sole nourishment during four days, he soon alleviated these symptoms. Ignorants give oil uselessly in such cases. As for me, I would prefer water to oil, and to all other liquids, for the reason that being easily taken in great amount, it seems better calculated to take up the particles of corrosive sublimate than all the other thicker liquids; for they are already impregnated with particles of other bodies." This fact, according to Dr. George, author of a work entitled "*Médecine des Campagnes à l' aide des substances usuelles*" (medicine of the country districts with additional means beyond the ones in general use,) proves that there is no substance, however caustic, that being diluted in a sufficient quantity of water, may not become inoffensive.

Water, then, attenuates the effect of poison and acts as a neutralizer. It fills, besides, the second indication of the treatment of poisoning, that which consists in promoting the elimination of the poison, whatever it may be. Thus this elimination is accomplished by four principal channels: the superior and inferior digestive orifices, the skin and the kidneys. But tepid water is emetic, cold water is diuretic. Why not then oftener resort than customary to this method, so simple and so happily put in practice by the learned Sydenham?

Water, remarks Dr. George, has the first virtue of an antidote applicable to all poisonous agents, and is available at all hours. We time and again read in the papers that a workman or other individual has swallowed, by mishap, a glass of aqua fortis, or a solution of copper, or a solution of sulphuric acid. While a physician is being hunted up, the poor fellow has died. In all these cases, absolute common water, cold or warm, taken in abundance, would suffice to neutralize the poison and save the victim.

The above is a means of saving life which cannot be made too public.—*N. O. Jour. of Medicine.*

